# CO _2_ Laser Technique versus Cold Steel: Is CO _2_ Laser Required as a Surgical Tool for Flawless Stapes Surgery?

**DOI:** 10.1055/s-0044-1801315

**Published:** 2025-01-22

**Authors:** Vikas Kumar, Anandita Gupta, A. Sethi

**Affiliations:** 1 Department of Otorhinolaryngology, Head and Neck Surgery, Army College of Medical Sciences, Brar Square, Delhi Cantonment, New Delhi, India

**Keywords:** laser stapedotomy, conventional stapedotomy, otosclerosis, comparison, hearing gain

## Abstract

**Introduction** Stapedotomy is the standard of care in the surgical management of clinical otosclerosis. It is a precise and technically demanding craft requiring impeccable surgical skills. Both conventional and laser-assisted procedures aim to achieve closure of the air-bone gap (ABG) with minimum collateral damage.

**Objective** To compare the postoperative outcomes of conventional stapes surgery and CO _2_ laser-assisted surgery.

**Methods** We conducted a retrospective analysis of the medical records of 74 adult patients who underwent stapedotomy at our center. The patients were divided into two groups for comparison: the “cold steel method” (CSM) group, which was composed of patients who had undergone conventional stapedotomy (manual microperforators/hand-held microdrill); and the “CO _2_ laser-assisted” (LA) group. The postoperative outcomes assessed at 3 and 6 months in both groups were analyzed and compared. The average operative time and complications of both groups were also compared.

**Results** The hearing outcomes presented statistically significant postoperative improvement in both groups. The LA group presented statistically significant better air conduction thresholds at 3 and 6 months ( *p* < 0.05). The ABG and its degree of closure were statistically better at 3 months in the LA group; however, the difference became insignificant at 6 months. Transient vertigo was more common in the LA group ( *p* < 0.01). There was no new sensorineural hearing loss in either group. The operative time was longer in the LA group.

**Conclusion** In experienced hands, both conventional and laser techniques can be used with equal ease and expectation of better outcomes. The CO _2_ laser is not an indispensable tool to achieve good surgical results on a routine basis.

## Introduction

Currently, total or partial stapedectomy has been replaced by small fenestra stapes surgery as the choice of treatment for otosclerosis (OS). The surgical techniques that can be used to perform a stapedotomy include hand-held microperforators, microdrill, various lasers, or a combination of these modalities in various steps, with the prerequisite being delicate handling of the stapes to minimize inner ear trauma.

The use of hand-held microperforators enables a controlled serial dilatation of the stapes fenestra. However, inadvertent fracture of the stapes footplate (SFP) due to the application of extra pressure is a possible risk. Moreover, in certain clinical situations in which the SFP is minimally fixed, excessive movement of the stapes while using the microperforators can damage the membranous labyrinth. The use of microperforators to fenestrate a minimally fixed annulus or a biscuit-type SFP (thick OS focus on the SFP without fixation of the annulus), can result in a floating footplate, a condition in which the SFP sinks in the vestibule, thereby predisposing to extensive inner ear damage. On the other hand, using a laser in these situations provides the advantage of atraumatic stapes handling. 1 Any degree of sensorineural hearing loss (SNHL) after the surgery is devastating to both the surgeon and the patient. The risk of developing SNHL has been reported to be lower than 2% in primary cases and 4% in revision cases. 2 3 4 The incidence of complete SNHL has been reported to be lower than 1% in stapedotomy. 2 However, a higher incidence, of 2.7%, has been reported in stapedectomy. 5 Serous labyrinthitis, extensive drilling resulting in acoustic trauma, perilymph gusher, traumatic handling of stapes, and excessive bleeding are some of the factors attributed to the causation of either transient or permanent SNHL and vertigo after stapes surgery. Irreversible morphological changes in the inner ear can occur because of inner ear damage due to these conditions. 5

Lasers such as argon, potassium titanyl phosphate (KTP), CO _2_ , and diode offer an expedient solution due to bloodless field, high precision, and possibility of fewer injuries to the inner ear and ossicular chain. 6 Currently, the preferred laser for stapedotomy is the CO _2_ laser, due to the availability of a micromanipulator that can be mounted on the microscope to direct the aligned beams with an accurate spot size on the SFP. Moreover, it gets readily absorbed by the stapes bone, resulting in lower levels of penetration and resultant damage to the deeper tissues. A very thin perilymph layer is sufficient to absorb most of the energy of the CO _2_ laser, thus protecting the underlying vestibular structures. An elevation of 0.3° C in the temperature of perilymph, as demonstrated in experimental studies by Lesinski 7 and Lesinski and Palmer, 8 is insufficient to damage the inner ear tissues. Further, any inadvertent risk of inner damage either due to the penetration of laser energy or due to the heating of the perilymph has been surmounted by the application of the CO _2_ laser as a “one-shot” using the scanner system at power settings of 1 W to 20 W, with a pulse duration ≤ 0.05 seconds in continuous mode. 9

The quest for a perfect tool to manipulate the stapes and create a small fenestra on the SFP without any collateral inner ear damage is still ongoing. The purpose of the present study is to compare the surgical outcomes, operative time, and complications of conventional stapedotomy using hand-held microperforators and CO _2_ laser-assisted stapes surgery.

## Methods

### Study Design

The present study is a retrospective chart review of adult patients (aged > 18 years) who were suspected of having clinical OS and who subsequently underwent stapedotomy at our tertiary care center between 2013 and 2019. The diagnosis of OS was based on clinical examination and diagnostic evaluation. The audiologic evaluation was performed by a national regulatory body-certified audiologist.

We extracted data extraction pertaining to: a) demographics; b) preoperative air conduction (AC) and bone conduction (BC) thresholds (averages for the AC and BC thresholds at 0.5, 1, 2, and 4 kHz were calculated); c) preoperative air-bone gap (ABG), which was calculated by subtracting 4 tone AC averages from 4 tone BC averages; d) post-operative AC and BC thresholds at 3 and 6 months (averages for the AC and BC thresholds at 0.5, 1, 2, and 4 kHz were calculated); e) postoperative ABG at 3 and six months, which was calculated by subtracting 4 tone AC averages from 4 tone BC averages; f) onset of new symptoms after surgery; and g) total operative time. Data on speech discrimination was not available for all patients; hence, it was not collected for analysis.

Patients with following parameters were included: a) ABG greater than 20 dB on pure-tone audiometry (PTA); b) surgical procedure consisting of conventional stapedotomy using manual microperforators/hand-held burr or CO _2_ laser-assisted stapedotomy; and c) age >18 years. Patients presenting the following characteristics were not included in the chart review: a) those who underwent stapedotomy assisted by electric microdrill (skeeter otologic drill, Xomed, Medtronic plc, Minneapolis, MN, United States); b) subjects with co-morbidities such as hypertension and diabetes mellitus; c) those with preoperative mixed hearing loss; d) individuals with postoperative follow up shorter than 6 months; e) those submitted to revision stapes surgery; and f) subjects with surgically-documented tympanosclerosis or attic fixation of the ossicles as a cause of conductive hearing loss.

The diagnosis was confirmed perioperatively by checking the mobility of the ossicular chain and the status of the middle ear mucosa (MEM) in all cases. The current study was approved by the institutional ethical committee.

### Surgical Procedure

All surgical procedures were performed under local anesthesia (2% lignocaine + 1:80 thousand adrenaline) supplemented with intravenous sedation (50 mcg fentanyl + 1 mg midazolam). Using the permeatal approach, a tympanomeatal (TM) flap was elevated from the 12 o'clock to the 6 o'clock position 5 mm to 6 mm lateral to the annulus. For adequate exposure, the posterosuperior bony canal wall was partially removed, either using a House curette (Karl Storz SE & Co. KG, Tuttlingen, Germany) or a micromotor drill. The chorda tympani nerve was reflected anteroinferiorly, and the subsequent steps included: a) checking the MEM and ossicular mobility, confirmation of stapes fixation; b) creation of the stapes fenestra; c) dislocation of the incudostapedial joint; d) sectioning of the stapedius tendon; e) and removal of the stapes suprastructure. The stapes fenestra was serially enlarged with the help of microperforators of 0.3 mm to 0.7 mm in size. The margins of the fenestra were smoothened with a hand-held microburr of 0.8 mm in size. Alternatively, the stapedotomy, the removal of the stapes superstructure, and the sectioning of the stapedius tendon were accomplished by CO _2_ laser (SurgiTouch, Lumenis Be Ltd., Yokneam, Israel) using the “one-shot” technique. The following parameters were used: power of 2 W to 5 W; pulse duration of 0.05 seconds; continuous wave mode; and spot size of 0.2 mm. For the stapedotomy, the power settings were increased to 15 W to 20 W. Additional shots were fired to transect the posterior crura of the stapes or to enlarge the stapedotomy, when required. The laser pulse was directed using a micromanipulator attached to the microscope.

Following stapedotomy, a Teflon-platinum piston (Grace Medical, Memphis, Tennessee, Estados Unidos) of 0.6 mm in diameter and 4.5 mm to 4.75 mm in length was placed and crimped onto the long process of the incus. The stapedotomy was sealed with fat harvested from the ear lobule, and the TM flap was repositioned. Subjective improvement in hearing was tested at the end of the procedure, followed by packing of the external auditory canal (EAC) with medicated Gelfoam (Pfizer Inc., New York, NY, United States). All patients received postoperative antibiotics (intravenous ceftriaxone 1 gm q 12 h) and steroids (intravenous dexamethasone 6 mg q 12 h) for 2 days. No labyrinthine sedatives were administered routinely.

### Data Analysis

For the purpose of analysis, the patients were divided into two groups: the “cold steel method” (CSM) group, which included patients who had undergone conventional stapedotomy using manual microperforators; and the “CO _2_ laser-assisted” (LA) group. The following parameters were analyzed: a) PTA air conduction thresholds and ABG before surgery; b) PTA air conduction thresholds and ABG 3 and 6 months after surgery; postoperative ABG in each group, which was classified as being within 0 dB and 10 dB, 11 dB and 20 dB, 21 dB and 30 dB, and > 30 dB; c) degree of AB gap closure at 3 and 6 months, which was calculated by subtracting the pre- and post-operative ABG; d) onset of new symptoms (severe vertigo, new onset SNHL); and e) total operative time.

The mean preoperative AC thresholds and ABG of the groups were compared using the Mann-Whitney U test to ensure homogeneity. The statistical analysis was conducted using the IBM SPSS Statistics for Windows (IBM Corp., Armonk, NY, United States) software, version 23.0. Values of *p* < 0.05 were considered significant at a 95% confidence level. All continuous variables were expressed as mean and standard deviation (SD) values.

## Results

### Population

Between January 2013 and December 2019 at our center, 41 patients underwent conventional stapes surgery and 33 underwent laser stapes surgery. After the application of the inclusion and exclusion criteria, we analyzed data from 30 patients in each group. The mean age of patients in the CSM group was of 36.23 (SD: ± 4.67) years, and of 39.17 (SD: ± 5.17) years in the LA group. There was no significant difference in terms of gender distribution between the groups ( *p* = 0.19). The demographics of the sample are shown in Table 1 .

**Table 1:** Demographics of patients included in the study

	CSM group (n = 30)	LA group (n = 30)	*p* -value
**Age in years: mean ± (standard deviation)**	36.23 (± 4.67)	39.17(± 5.17)	0.03
**Gender: n (%)**			
Male	19 (63%)	14 (47%)	0.19
Female	11 (37%)	16 (53%)	
**Laterality: n**			
Right	16	17	
Left	14	13	

**Abbreviations:** CSM cold steel method; LA, CO _2_ laser-assisted.

**Note:** Values of *p* < 0.05 were deemed significant.

### Preoperative Hearing Status

The mean preoperative audiometric AC thresholds and ABG in both groups are shown in Table 2 . There was no statistically significant difference for either of the preoperative parameters ( *p* > 0.05) between the two groups.

**Table 2:** Preoperative audiometric data

	CSM group (n = 30)	LA group (n = 30)	*p* -value
**Air conduction thresholds: mean ± (standard deviation)**	49.2(± 5.94)	49.73(± 5.56)	0.72
**Air-bone gap: mean ± (standard deviation)**	36.9(± 5.57)	37.17(± 4.48)	0.84

**Abbreviations:** CSM cold steel method; LA, CO _2_ laser assisted.

**Note:** Values of *p* < 0.05 were deemed significant.

### Postoperative Hearing Outcomes

Successful surgical outcomes were documented in both groups ( Tables 3 4 ). All the parameters were indicative of postoperative improvement in both groups. Table 4 summarizes the postoperative improvement in audiometric parameters, which were as follows.

**Table 3:** Comparison of pre and postoperative audiometric data between the study groups

	CSM group (n = 30)		*p* -value	LA group (n = 30)		*p* -value
	Pre-operatively	Post-operatively (6 months)		Preoperatively	Postoperatively (6 months)	
**Air conduction thresholds: mean ± (standard deviation)**	49.2(± 5.94)	22.13(± 4.29)	< 0.01	49.73(± 5.56)	19.77(± 3.09)	< 0.01
**Air-bone gap: mean ± (standard deviation)**	36.9(± 5.57)	10.73(± 3.54)	< 0.01	37.17(± 4.48)	10(± 1.97)	< 0.01

**Abbreviations:** CSM cold steel method; LA, CO _2_ laser assisted.

**Note:** Values of *p* < 0.05 were deemed significant.

**Table 4:** Postoperative audiometric data, complications, and operative time

	CSM group (n = 30)	LA group (n = 30)	*p* -value
**Air conduction threshold: mean ± (standard deviation)**			
3 months	23.1(± 4.48)	20.53(± 3.55)	0.02
6 months	22.13(± 4.29)	19.77(± 3.09)	0.012
**Air-bone gap: mean ± (standard deviation)**			
3 months	11.4(± 3.82)	10.2(± 2.31)	0.015
6 months	10.73(± 3.54)	10(± 1.97)	0.33
**Air-bone gap closure: mean ± (standard deviation)**			
3 months	25.5(± 5.28)	27.03(± 5.8)	0.029
6 months	26.16(± 5.77)	27.3(± 5.63)	0.44
**Air-bone gap within 0-10 dB: (%)**			
3 months	63%	60%	0.79
6 months	63%	67%	0.79
**Operative time in minutes: mean ± (standard deviation)**	89.33(± 5.49)	107.6(± 4.18)	< 0.01
**New onset of vertigo: (%)**	7%	33%	< 0.01

**Abbreviations:** CSM cold steel method; LA, CO _2_ laser-assisted.

**Note:** Values of *p* < 0.05 were deemed significant.

a) **AC thresholds** – Overall, there was improvement in mean the postoperative AC thresholds in both groups when compared to the pre-operative values ( *p* < 0.01). However, the results were better in the LA group both at 3 and 6 months of follow-up. This difference was statistically significant at 3 ( *p* = 0.02) and 6 months ( *p* = 0.012).
b) **ABG** – The mean ABG reduced in both groups ( *p* < 0.01) after surgery. It was better for the LA group at 3 months, with the difference being statistically significant ( *p* = 0.015); however, at 6 months, the difference was not statistically significant ( *p* = 0.33).
c) **Degree of ABG closure** – Calculated by subtracting the pre- and postoperative mean ABG, it was higher in LA group at 3 and 6 months postoperatively. This difference was statistically significant at 3 months ( *p* = 0.029), but not at 6 months ( *p* = 0.44).
d) **ABG within 0 dB to 10 dB, 11 dB to 20 dB, and 21 dB to 30 dB** – At 3 months, 63% of the patients in the CSM and 60% of the patients in LA group had achieved ABG closure within 0 dB and 10 dB ( *p* = 0.79); 33% of the patients in the CSM group and 40% of those in the LA group achieved ABG closure within 11 dB and 20 dB ( *p* = 0.59); and only 1 patient in CSM group had ABG closure within 21 dB and 30 dB. At 6 months, 63% of the CSM patients continued to have ABG within 0 dB and 10 dB, as well as 67% of the LA patients ( *p* = 0.79); 37% of the CSM patients and 33% of the LA patients continued to have ABG closure within 11 dB and 20 dB; and no patient in either group was documented to have ABG within 21 dB and 30 dB at 6 months.

### Operative Time

**Fig. 1:**
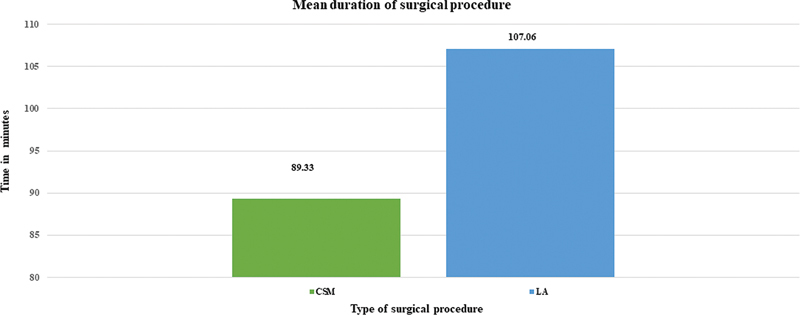
Difference in operative time between the study groups.

The mean operative time in the CSM group was of 89.33 (SD: ± 5.49) minutes, and in the LA group, it was of 107.06 (SD: ± 4.18) minutes, and this difference was statistically significant ( *p* < 0.01). The calculation of the operational time included the setting up of the laser and its associated precautions in the operating room (OR) ( Fig. 1 ).

### Complications

Complications were uncommon among the whole sample. No new onset of SNHL was documented in either group. New onset of vertigo requiring initiation of drug therapy was observed in 33% of the LA patients, and only in 7% of the CSM patients ( *p* < 0.01) ( Table 4 ). In both groups, the vertiginous symptoms subsided by the third postoperative day.

## Discussion

In the current study, we compared the hearing outcomes, postoperative complications, and operative time of conventional stapedotomy (with hand-held microperforators) and CO _2_ laser-assisted stapedotomy. Both groups presented postoperative improvement in the mean AC thresholds and reduction in ABG at 3 and 6 months. In both groups, ≥ 60% of the patients achieved ABG < 10 dB. However, the postoperative mean AC thresholds, the mean ABG, and the degree of ABG closure were statistically significant in favor of the LA group at 3 months. While the mean AC thresholds continued to be statistically better for the LA group, the other parameters became statistically insignificant at 6 months. None of the patients in the sample presented obliterative OS, obviating the use of the microdrill to create the SFP fenestra.

There is much evidence in the literature favoring the use of CO _2_ laser to achieve better surgical results and fewer complications. 10 11 12 The creation of a small fenestra in the stapes footplate is a very delicate and precise step. The transfer of energy into the inner ear during perforation of the SFP can be catastrophic, as it can result in cochlear and vestibular damage. The implications can vary from partial or total loss of residual hearing to severe vertigo and tinnitus. The CO _2_ laser is now preferred due to the availability of a micromanipulator that can be mounted on the microscope to direct the aligned beams with an accurate spot size on the SFP. Moreover, it gets readily absorbed by the stapes bone, resulting in lower levels of penetration and resultant damage to the deeper tissues. A very thin perilymph layer is sufficient to absorb most of the energy of the CO _2_ laser, thus protecting the underlying vestibular structures. 7 8 Further, any inadvertent risk of inner damage either due to the penetration of laser energy or due to the heating of the perilymph has been surmounted by the application of the CO _2_ laser as a “one-shot” using the scanner system at power settings of 1 W to 20 W, with a pulse duration of ≤ 0.05 seconds in continuous mode. 9 The CO _2_ laser is a perfect tool to precisely perform stapedotomy, irrespective of the degree of fixation and SFP thickness. Some of the inherent advantages of the CO _2_ laser are atraumatic stapes handling and avoidance of any inadvertent SFP fracture during manipulation. Moreover, in certain conditions, such as a minimally fixed annulus or a biscuit-type SFP (thick OS focus on the SFP without fixation of the annulus), the risk of developing a floating footplate, a condition in which the SFP sinks in the vestibule with resultant extensive inner ear damage, is significantly reduced by using the CO _2_ laser. 1

By evaluating the outcomes of conventional stapedotomy and laser-assisted stapedotomy, we were able to assess the usefulness of the CO _2_ laser for the surgical management of OS at our center. When comparing the complications, no untoward risk of SNHL could be demonstrated in the LA group, which is in line with the available literature. 10 11 Transient vertigo requiring medication was, however, more common in the LA group, which is in contradiction to the results published by Motta and Moscillo. 10

As ≥ 60% of the patients achieved ABG < 10 dB in both groups, there was no overwhelming advantage in using the CO _2_ laser, especially when considering the cost factor. Use of the CO _2_ laser significantly increases the overall cost of the surgical procedure. 13 14 Apart from the cost of CO _2_ laser equipment, increased use of the OR to set up the laser equipment and its associated precautions should be factored in. Moreover, handling of the laser equipment requires a certain level of expertise and, therefore, training of the OR staff.

Institutions where the CO _2_ laser and technical expertise are routinely available may use it to their advantage. In State-subsidized health care systems, especially in developing nations, the routine use of the CO _2_ laser does not offer any formidable advantage in terms of surgical results and reduction in the complication rate. On the contrary, increased operative time and OR use, as observed in the current study, may be a deterrent in advocating the use of the CO _2_ laser while performing routine stapedotomies. In our experience, only two situations, when encountered perioperatively, warrant a mandatory detour from the conventional stapedotomy: presence of obliterative OS, which can be documented in high-resolution computed tomography (HRCT) and warrants use of micromotor drill; and an incompletely fixed annulus or a biscuit-type OS involving the SFP, with a propensity for floating footplate, which can be safely handled by using the CO _2_ laser.

The current study has limitations inherent to a retrospective study. The sample size was small, due to the limited number of patient records satisfying the inclusion criteria. The present study suffers from multiple testing. A prospective study would yield more reliable conclusions about the relative efficacy of the two surgical procedures.

## Conclusion

According to our findings, there is a marginal advantage to using the CO _2_ laser to improve the surgical results without increasing the permanent complications. However, these results were achieved at the cost of increased operative time and expenses. In our view, surgeons can use the CO _2_ laser to their advantage while performing critical steps to increase the transmission of vibrations into the inner ear on a case-by-case basis. The CO _2_ laser is not an indispensable tool to achieve good surgical results on a routine basis. Finally, good postoperative hearing outcomes are more dependent on the otologist's impeccable technique and well-practiced surgical steps than on a specific tool.
